# Diagnostic criteria for musculoskeletal disorders for use in occupational healthcare or research: a scoping review of consensus- and synthesised-based case definitions

**DOI:** 10.1186/s12891-021-04031-z

**Published:** 2021-02-11

**Authors:** Henk F. van der Molen, Steven Visser, Jose Hernán Alfonso, Stefania Curti, Stefano Mattioli, David Rempel, Yves Roquelaure, P. Paul F. M. Kuijer, Sietske J. Tamminga

**Affiliations:** 1 Department of Public and Occupational Health, Coronel Institute of Occupational Health, Netherlands Center for Occupational Diseases, Amsterdam Public Health Research Institute, Amsterdam UMC, University of Amsterdam, Meibergdreef 9, Amsterdam, the Netherlands; 2 Department of Occupational Medicine and Epidemiology, National Institute of Occupational Health, Oslo, Norway; 3 Department of Medical and Surgical Sciences, University of Bologna, Bologna, Italy; 4 Division of Occupational and Environmental Medicine, University of California, San Francisco, USA; 5 Univ Angers, CHU Angers, Univ Rennes, Inserm, EHESP, Irset (Institut de recherche en santé, environnement et travail), UMR_S 1085, F-49000 Angers, France

**Keywords:** Case definition, Low back pain, Lumbosacral radicular syndrome, Subacromial pain syndrome, Carpal tunnel syndrome, Lateral or medial elbow tendinopathy, Epicondylitis, Knee osteoarthritis, Hip osteoarthritis, Occupational disease, Occupational healthcare

## Abstract

**Background:**

The aim of this study was to identify case definitions of diagnostic criteria for specific musculoskeletal disorders (MSDs) for use in occupational healthcare, surveillance or research.

**Methods:**

A scoping review was performed in Medline and Web of Science from 2000 to 2020 by an international team of researchers and clinicians, using the Arksey and O’Malley framework to identify case definitions based on expert consensus or a synthesis of the literature. Seven MSDs were considered: non-specific low back pain (LBP), lumbosacral radicular syndrome (LRS), subacromial pain syndrome (SAPS), carpal tunnel syndrome (CTS), lateral or medial elbow tendinopathy, and knee and hip osteoarthritis (OA). Case definitions for occupational healthcare or research were charted according to symptoms, signs and instrumental assessment of signs, and if reported, on work-related exposure criteria.

**Results:**

In total, 2404 studies were identified of which 39 were included. Fifteen studies (38%) reported on non-specific LBP, followed by knee OA (*n* = 8;21%) and CTS (*n* = 8;21%). For non-specific LBP, studies agreed in general on which symptoms (i.e., pain in lower back) and signs (i.e., absence of red flags) constituted a case definition while for the other MSDs considerable heterogeneity was found. Only two studies (5%), describing case definitions for LBP, CTS, and SAPS and lateral and medial elbow tendinopathy respectively, included work-related exposure criteria in their clinical assessment.

**Conclusion:**

We found that studies on non-specific LBP agreed in general on which symptoms and signs constitute a case definition, while considerable heterogeneity was found for the other MSDs. For prevention of work-related MSDs, these MSD case definitions should preferably include work-related exposure criteria.

**Supplementary Information:**

The online version contains supplementary material available at 10.1186/s12891-021-04031-z.

## Background

The accurate assessment of work-related etiological factors related to the onset or worsening of musculoskeletal disorders (MSDs) and diseases is acknowledged as an important research task to provide evidence base for clinical decision-making regarding occupational prevention and return to work [1]. Progress on understanding etiological factors related to the onset or worsening of work-related MSDs may, however, be hampered by variation in MSD case definitions and how these are assessed in occupational cohort studies [2]. As reported by Verbeek in 2012, a case definition consists of a minimum set of symptoms, signs and other data that are needed to establish a diagnosis [3], which can be used in clinical care, health surveillance or research. This variation in case definitions hinders comparison between studies and prevents the combining of studies in meta-analyses. The Network on the Coordination and Harmonisation of European Occupational Cohorts (OMEGA-NET) is an European Cooperation in Science and Technology (EU COST) action aimed at optimising the use of occupational cohorts at the European level [4]. One of its goals is to harmonise occupational exposure and health outcome information. For this reason, we aimed to harmonise case definitions of MSDs in occupational cohort studies.

Another similar initiative has been active since 2018. The Scientific Committee on work-related MSDs of the International Commission of Occupational Health (ICOH) was tasked with developing international consensus criteria for the clinical assessment of work-related MSDs [5, 6]. This initiative began with the recruitment of international experts, followed by the development of an overall framework for such criteria and the application of the framework for specific MSDs. The aim is to update and revise for example the criteria document for evaluating the work-relatedness of upper-extremity musculoskeletal disorders [7] and non-specific low back pain [8, 9]. Ultimately, this will lead to a publication on the framework and the publication of consensus criteria for prevalent MSDs.

However, it is currently not known how many MSDs have formulated case definitions and whether there is much variation between these disorder-specific case definitions. At the same time, it is also not known whether work-related exposure criteria are included in these MSD case definitions, if applicable. Consensus regarding case definitions may be agreed upon for example in a formal clinical guideline development process or be based on a Delphi technique, while a synthesis of the literature on case definitions may be based on a systematic review. Since our aim is to scope the literature on case definitions for use in both clinical care as well as for research - an appropriate approach would be to provide an overview of the literature using a scoping review. A systematic review process, which aims to answer a narrow research question, was deemed too limited for our research questions [10]. Performing a scoping review of the literature on criteria for MSDs would also support the ICOH Scientific Committee on MSDs in the development of an overall framework for consensus criteria regarding the occupational clinical assessment of work-related MSDs. It might also be used as a basis for the OMEGA-NET in reaching consensus on case definitions used in occupational cohort studies.

Specifically, this scoping review aimed to identify case definitions of diagnostic criteria for specific musculoskeletal disorders (MSDs) for use in occupational healthcare, surveillance or research. For the purpose of this scoping review, we included prevalent MSDs and diseases that have been reported to be work-related: non-specific low back pain (LBP), lumbosacral radicular syndrome (LRS), subacromial pain syndrome (SAPS), carpal tunnel syndrome (CTS), elbow tendinopathies, and knee and hip osteoarthritis (OA) [11–17]. Some of the MSDs included can be considered diseases (‘a particular distinctive process in the body with a specific cause and characteristic symptoms’) such as osteoarthritis while other MSDs included can be considered disorders (‘irregularity, disturbance, or interruption of normal functions’) such as LBP. However, to improve readability, musculoskeletal disorder (MSD) is used throughout this scoping review.

## Methods

A scoping review was performed using the Arksey and O’Malley framework [10], which is characterised by a research question that usually leads to the inclusion of studies encompassing various study designs and charting the data in tables/figures. Such reviews do *not* synthesise the data *or* perform a quality assessment of the studies included but provide an overview of the literature [10]. In addition, the checklist of Preferred Reporting Items for Systematic Reviews - extension for Scoping Reviews (PRISMA-ScR) was used [18]. A protocol was established before beginning this scoping review, although it was not registered.

### Study selection

#### Data sources and search terms

We systematically searched the electronic database of Medline and Web of Science for studies between 2000 and 26 June 2020. Because this scoping review serves as a basis for further research leading to the development of an overall framework for consensus criteria regarding the clinical assessment of work-related MSDs and for reaching consensus on case definitions used in occupational cohort studies, we were only interested in recent insights. The specific search strategy is described in Additional file 1.

#### Article selection

To be included in this scoping review, a study had to: (i) include diagnostic criteria for any of the following MSDs: non-specific LBP, LRS, SAPS, CTS, lateral or medial elbow tendinopathy, or knee or hip OA; (ii) report diagnostic criteria that were based on a consensus- or synthesised-based method; (iii) report a description of MSD diagnostic criteria in terms of symptoms, signs and/or instrumental assessment of signs, e.g. Magnetic Resonance Imaging (MRI), and (iv) full text be available in English, Spanish, German, French, Norwegian, Swedish, Danish, Dutch or Italian. Articles were also included even when work-related exposure criteria were not part of the case definition.

Article selection was performed in three steps. First, titles and abstracts were independently screened by pairs of authors (HvM, JHA, SV, SC, PK, SM and ST) to identify potentially relevant studies by means of the online software tool Rayyan [19]. Second, the full texts of potentially relevant studies were independently assessed for eligibility against the inclusion criteria by the same pairs of authors. Disagreement between these authors about the inclusion of studies occurred for about 6% (135/2404) of the articles screened on title and abstract and for about 43% (44/102) of the articles screened on full text. Disagreement about the inclusion based on title and abstract and full text was resolved through in-depth face to face discussion mostly on the fact whether the study really used a consensus- or synthesised based method by HvM, SV and ST.

#### Charting the data

A description of the article, the aim of the study (i.e., case definition for clinical practice or research), type of MSD, method (i.e., expert consensus, guideline based on systematic literature review or synthesis of the literature), the case definition of MSD and, if reported, work-related exposure criteria were charted by two authors (SV and ST) on a pre-designed data-charting form. Subsequently, each author checked 100% of the data charting by the other, which consensus was reached by discussion. Case definitions were collated in accordance with Violante and colleagues (2019) [1, 20]: (i) symptoms, (ii) signs and (iii) instrumental assessment of signs.

## Results

### Selected studies

**Fig. 1:**
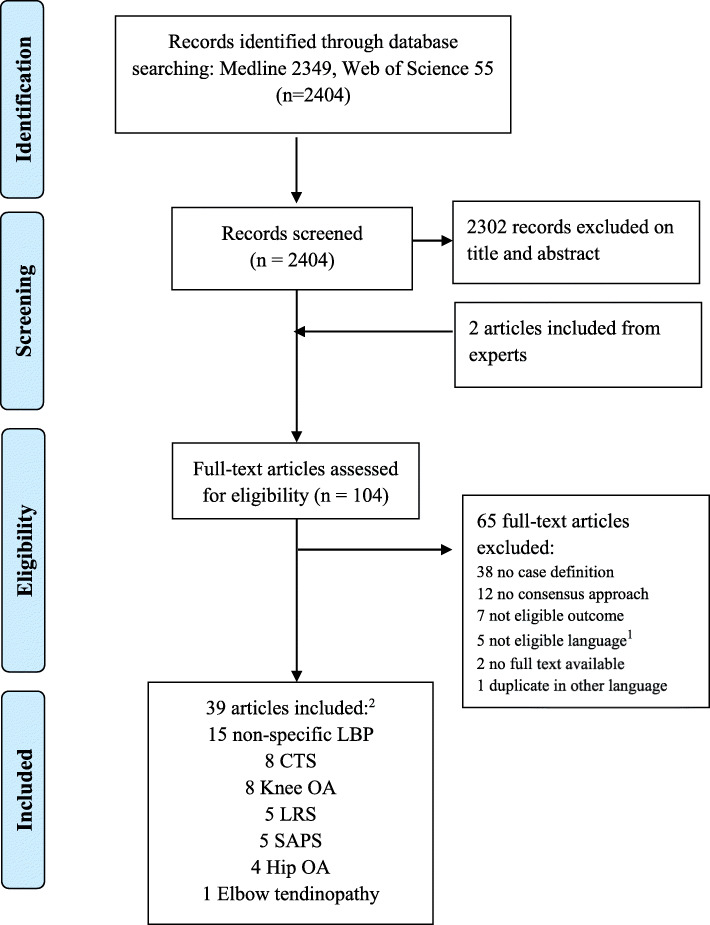
PRISMA Flow diagram. Abbreviations: LBP (Low Back Pain); CTS (Carpal Tunnel Syndrome); OA (Osteoarthritis). LRS (Lumbosacral Radicular Syndrome); SAPS (Subacromial Pain Syndrome); 1. Full-text not available in English, Spanish, German, French, Norwegian, Swedish, Danish, Dutch or Italian. 2. Numbers do not add up as some articles included various diseases/disorders

A PRISMA flow diagram of the study selection process is shown in Fig. 1. In total, 2404 references were retrieved from the two databases and assessed on title and abstract. The full texts of 104 potentially eligible articles were then examined, of which 39 studies met the inclusion criteria, of which 2 were included from experts. Reasons for excluding studies based on full text are presented in Additional file 2.

### Study characteristics

In total, 39 articles [7, 13, 21–57] described diagnostic criteria for MSD case definitions for non-specific LBP (*n* = 15, 38%) [25, 27, 29, 30, 37–41, 44, 47–49, 52, 54], LRS (*n* = 6, 15%) [26, 32, 37, 40, 47, 56], SAPS (*n* = 5, 13%) [7, 13, 21, 31, 53], CTS (*n* = 8, 21%) [7, 23, 24, 33, 34, 36, 50], lateral and medial elbow tendinopathy (*n* = 1, 3%) [7], and knee (*n* = 8, 21%) [22, 35, 42, 43, 45, 46, 55, 57] and hip OA (*n* = 4,10%) [42, 45, 51, 55]. The charting of these articles is presented in Additional file 3.

Most studies were conducted in Europe (*n* = 21, 54%) [7, 13, 21, 23, 25, 26, 32, 33, 37–41, 45, 47, 50–52, 55–57], reported a synthesis of the literature on case definitions (*n* = 15, 38%) [7, 21, 22, 24, 27, 28, 36, 39, 41, 46, 48–51, 57], and/or had a clinical aim (*n* = 32, 82%) [13, 21–27, 31–41, 43–50, 52, 53, 55–57], while almost half of the studies reported three or more disciplines that were involved in reaching expert consensus regarding the case definition (*n* = 19, 49%) [13, 21, 24–26, 29, 31, 32, 37, 41–43, 45–48, 52, 55, 56] (Additional file 3).

### MSD case definitions

Regarding non-specific LBP (Table 1, Additional file 3), there was general agreement between the studies included that diagnostic criteria for non-specific LBP should consist of pain in the low back without leg pain and absence of specific pathology of the LBP [25, 27, 30, 37–41, 47–49, 52]. Some of the studies referred to a specific duration of the symptoms, which varied from < 1 month to < 12 weeks [30, 37, 38, 40, 47]. Most studies also agreed that imaging should *not* be recommended [25, 27, 38–40, 49, 52]. The same was true for LRS with the specification that leg pain should be reported [26, 29, 32, 39, 40, 47, 54, 56]. Additionally, the same was found for *chronic* LBP, with the duration of pain in the low back referring to chronicity varying from > 6 weeks to > 12 weeks [37–41, 44, 47, 49].

**Table 1:** Collating of all reported symptoms, signs and instrumental assessment of signs of the 39 included studies on MSD case definitions

MSD category		Symptoms	Signs	Imaging
Non-specific LBP	Acute	➣ Pain in low back [24, 26, 29, 38, 47, 48, 51] < 1 month [46] to < 12 weeks [36, 37, 39, 40]. ➣ Muscle tension or stiffness in lower back [36, 39]. ➣ Posterior irradiation not below the knee [46].	➣ No “red flags” (e.g. history of cancer, steroid use, fractures, infections) [24, 26, 36–39, 47, 48, 51]. ➣ Neurological examination (Lasègue’s test and crossing Lasègue’s test) [40].	Not recommended [24, 26, 37–40, 48, 51].
	Chronic	➣ Pain in low back > 12 weeks [28, 37–40, 43, 46] ➣ Recurrent: > 2 on an 11 point NRS for at least 24 h following a period of at least 30 days pain free [53]. ➣ Muscle tension or stiffness in lower back [36, 39]. ➣ Posterior irradiation not below the knee [46].	➣ No “red flags” (e.g. history of cancer, steroid use, fractures, infections) [36–39, 43, 48] ➣ Neurological examination (Lasègue’s test and crossing Lasègue’s test) [40].	Not recommended [37–40, 43, 48].
LRS		➣ Pain in low back [38, 39] < 1 month [46]. ➣ Muscle tension or stiffness or weakness in lower back [39, 55]. ➣ Posterior irradiation below the knee or anterior to the thigh [46]. ➣ Radicular pain in 1 lower limb [25, 31, 55].	➣ One or more positive neurological test indicating nerve root irritation or neurological deficit (e.g. a positive Lasègue’s test at 60°) [25, 31, 38, 39, 55]. ➣ Finger-floor distance of > 25 cm [55]. ➣ Neurological signs (e.g. incontinence) [55].	Not recommended [39, 55].
SAPS		➣ Shoulder pain [7, 13, 20, 30] worsened by active elevation [7, 13]. ➣ Weakness of shoulder muscles [20, 30]. ➣ Stiffness of shoulder joint [30]. ➣ Loose or unstable shoulder [30]. ➣ Painful clicking, grinding or clunking in the shoulder [30].	➣ Positive (pain or weakness) on one or more specific tests (e.g. Neer’s sign test or Painful arc test) [7, 13, 20, 30]. ➣ Retraction of tendon(s) to the glenoid rim, measured in either the coronal or axial plane, and/or ≥ 67% of the greater tuberosity exposed, measured in the sagittal plane, diagnosed either with MRI or intraoperatively [52].	Useful after 6 weeks of symptoms [13]. MRI [52]
CTS		➣ (Nocturnal) numbness of digits I, II or III [7, 22, 32, 33, 35, 49]. ➣ Pain in hand, wrist and forearm [7, 49]. ➣ Weakness/atrophy of thenar musculature [33]. ➣ Tingling feelings in digits I, II or III [22, 49].	➣ Positive on provocative tests (e.g. Tinel’s sign or Phalen’s test) [7, 33, 35]. ➣ Nerve conduction examination of the median nerve [22, 27, 35, 49]: a. Sensitive neurography: > 8 m/s compared with ulnar nerve [22] b. Distal motor latency: > 4.2 ms compared with ulnar nerve [22]. ➣ Cut off > 8,5 mm2 of median nerve cross-sectional area [23].	NR
Elbow tendinopathy: Lateral or medial		➣ Activity dependent pain around the lateral or medial epicondyle [7].	➣ Local pain on resisted wrist extension (lateral) or on resisted wrist flexion (medial) [7].	NR
OA	Hip	➣ Hip pain [41, 44, 50, 54]. ➣ Restricted range of motion of the hip [50]. ➣ Morning stiffness < 1 h in hip [50].	➣ Joint space narrowing [44, 50, 54]. ➣ Kellgren-Lawrence grade ≥ 2 [41]. ➣ Osteophytes [44, 54].	X-Ray [41, 44, 50, 54].
	Knee	➣ Knee pain [21, 34, 41, 42, 44, 45, 54, 56]. ➣ Morning stiffness < 30 min in knee [21, 34, 42, 54, 56]. ➣ Crepitus in knee [21, 34, 42, 54, 56]. ➣ Restricted range of motion of the knee [56].	➣ Bone enlargement or osteophytes [21, 44, 54, 56]. ➣ No palpable warmth [21, 54]. ➣ Joint space narrowing [44]. ➣ Synovial fluid; clear and viscous; leukocyte count < 2000/ml [21, 34]. ➣ Kellgren-Lawrence grade > 0 [45] or ≥ 2 [41, 42].	X-Ray [21, 34, 41, 42, 44, 45, 54, 56].

*Abbreviations*: *MSD* (Musculoskeletal Disorder), *LBP* (Low Back Pain), *NRS* (Numeric Rating Scale), *LRS* (Lumbosacral Radicular Syndrome), *SAPS* (Subacromial Pain Syndrome), *MRI* (Magnetic Resonance Imaging), *CTS* (Carpal Tunnel Syndrome), *OA* (Osteoarthritis)

The five studies included on SAPS reported that pain in the shoulder in combination with results from tests are needed to identify a patient with SAPS (Table 1, Additional file 3) [7, 13, 21, 31] and that retraction of tendon(s) to the glenoid rim indicate a massive rotator cuff tear [53]. The articles varied on which tests were needed (e.g., painful arc test, Neer’s sign test, Hawkins-Kennedy test, empty can, lift-off and drop arm test) and varied on the use of imaging. For example, some studies were of the opinion that X-ray was needed to assess degenerative changes, while others were of the opinion that ultrasound was needed to exclude a rotator cuff rupture.

More variation was found between the studies included on CTS (Table 1, Additional file 3) [7, 23, 24, 28, 33, 34, 36, 50]. Signs varied from none to numbness, pain, tingling, paraesthesia, brachialgia, and nocturnal numbness/paraesthesia in the fingers or hand and/or a combination of these. Determination of symptoms varied from conducting provocation tests such as Tinel’s sign and Phalen’s test [7, 34, 36], to *not* conducting these tests [33], or the assessment of nerve conduction speed, nerve cross-sectional area, and/or latency. Recommended imaging included electrodiagnostic and ultrasound to determine the cross-sectional area, while MRI was *not* recommended [23, 24, 36, 50].

The only article included on lateral and medial elbow tendinopathy showed that the symptom pain should be directly located around the lateral or medial epicondyle [7]. The pain should be intermittent and activity dependent. Furthermore, the pain should be present at assessment or on at least 4 days during the last 7 days. The signs of lateral elbow tendinopathy were reported as local pain on resisted wrist extension and the signs of medial elbow tendinopathy were reported as resisted wrist flexion (Table 1, Additional file 3).

All of the studies included on hip OA reported hip pain with three out of four of the studies reporting hip pain combined with joint space narrowing. All recommended X-ray to assess degeneration using the Kellgren-Lawrence scale (Table 1, Additional file 3) [42, 45, 51, 55]. More variation was found on knee OA. Symptoms ranged from knee pain to knee pain combined with stiffness, reduced function, swelling, cracking and/or grinding movement [22, 35, 42, 43, 45, 46, 55, 57]. Signs ranged from none to an extensive list of alterations in the knee, such as bony enlargement. Five of the eight studies on knee OA agreed on using X-ray to assess degeneration using the Kellgren-Lawrence scale [22, 35, 42, 43, 45, 46].

### Work-related exposure criteria

In one study [44] discussing chronic LBP the following working exposure criteria were reported: long-term spinal heavy burden, excessive rotation, or vibration.

In another study [7] discussing case definitions for SAPS, CTS and lateral and medial elbow tendinopathy, work-related exposure criteria were included in the clinical assessment of the case definition. Work-related exposure criteria for SAPS included: 1) postures: i) with hands behind the back, ii) where hand reaches to opposite part of the trunk, iii) with extreme rotation, iv) where the arm is unsupported by the body for several minutes; 2) elevation movements of the upper arm in comparison with those of the trunk; 3) high repetitions of movements of the upper extremity; and 4) a combination of more than average force and one of the movements or postures mentioned.

For CTS, work-related exposure criteria included: extreme wrist postures, handling (vibrating) tools, high repetition of wrist movements, high forces for the hands, and combination of postures/movements and forces [7].

Finally, for lateral and medial elbow tendinopathy work-related exposure criteria included: extreme flexion of the elbow, posture with extended elbows, posture with extreme pronation or supination of the elbow, high repetition of movements of the elbow, grasping or lifting of objects with high forces and/or combination of postures, movements and forces [7].

## Discussion

In this scoping review, we found that studies on non-specific LBP, agreed in general on which symptoms (i.e., pain in lower back) and signs (i.e., absence of red flags) constitute a case definition, while considerable heterogeneity was found for the other MSDs. Only two studies, describing case definitions for CTS, SAPS and lateral and medial elbow tendinopathy, included work-related exposure criteria in their clinical assessment.

### Comparison with the literature and recommendations for future research

While this scoping review identified various clinical and research settings in which consensus has been reached or the literature has been synthesised regarding case definitions, some knowledge gaps were found. Case definitions of diagnostic criteria for MSDs may differ depending on the setting and purpose. A setting may be a clinical or an occupational epidemiological research context, while the purpose may consist of prevention (including screening activities), prognosis or treatment. When considering a clinical setting we agree with Genevay et al. that case definitions for clinical practice require high sensitivity and high specificity, while case definitions for occupational epidemiological research mainly require higher specificity. High specificity is required, given that the inclusion of false positive cases need to be avoided in occupational epidemiological research [32] as it may influence the identification of personal- and work-related risk factors. However, prevention-related studies among workers may require just higher sensitivity for precautious reasons.

Case definitions for research and for workers’ health surveillance also need to consider practicalities such as costs, the burden of completing questionnaires, and the availability of resources. This need for balance resulted in some studies providing a minimal and an optimal case definition depending on its research purpose [30]. For example, Dionne et al. reported a minimal case definition consisting of two questions, one on back pain and one on severity, and an optimal case definition that was based on the minimal definition with additional questions covering frequency, duration of symptoms and severity, as well as a question covering sciatica and a question excluding other causes [30].

Case definitions aiming to assess the work-relatedness of a disorder/disease in association with workers’ health surveillance [58] or financial compensation may need higher sensitivity, given the financial costs involved. In both situations, a symptom questionnaire with high disorder/disease sensitivity and a follow-up medical examination may be the best option [58].

Considering the purpose of the case definition, those aimed at prevention in clinical care or research could encompass greater heterogeneity since sensitivity is less important given that this heterogeneity has a limited effect on identifying personal and work-related risk factors [59, 60]. For treatment at an individual level, a higher sensitivity is needed, especially in the case of high costs, detrimental side effects or the limited availability of resources. This is reflected, for example in the fact that case definitions used in a clinical care more often included imaging (Additional file 3).

Work-related exposure criteria in the clinical assessment of a case definition require more attention in future research [61, 62]. We only found two studies that reached consensus for work-related MSDs diagnostic case definitions [7, 44], although recently there are more studies available that address work-related risk factors for specific MSDs (e.g., [2, 11, 28, 63–67]). The International Classification of Diseases (ICD-11) of the World Health Organisation (WHO) [68] includes expert-based criteria for work-related diseases [68, 69]. These expert-based criteria, in combination with the data charted in this scoping review, could serve as a basis for a Delphi study aiming for the harmonisation of these case definitions, focussing on research aimed at prevention of work-related disorders/diseases.

In comparison with previous research on expert consensus methods in other fields such as medical education, we found that consensus methods were not standardised and not transparently reported; for example, no detailed data were provided about the participating experts [70]. Furthermore, we also found those studies which reported on a consensus method, most often did not provide a definition of consensus a priori, in terms of envisioned content and format of the intended outcome [70, 71]. Finally, previous research found that rating scales influence the outcome of these consensus methods [72] but less is known about which factors, such as tacit professional collective knowledge, affect the outcome of these consensus methods among experts. To that end, more research is needed on consensus methods themselves as well as criteria to improve the reporting of these consensus methods [71]. When that is achieved, future reviews on this topic might be able to only include high quality studies.

### Strengths and limitations

One strength of this review is that it encompassed a variety in reported consensus or synthesis approaches providing an overview of case definitions for the MSDs included, as reported in peer-reviewed medical journals. This appeared especially important for our research question, as we found that heterogeneous study designs were used to reach consensus or to synthesise the literature on case definitions. Another strength was the international research team of whom most have been involved in these case definition developments. Four limitations should be noted. First, we may have overlooked some relevant studies, since our search strategy was limited to one database and the Web of Science. Second, we may have overlooked some relevant studies since in some studies it was arbitrary whether or not the authors applied a consensus- or synthesized-based method. Third, in a scoping review, quality assessments are not performed, because the literature is charted without critical appraisal of the studies included [10]. However, additional file 3 shows that the studies included differed in the amount of information they provided regarding their research methods. In addition, it was not always made clear whether consensus was based on the consultation of experts from various disciplines, although the involvement of multiple disciplines was considered an essential element in reaching consensus regarding case definition [73]. Finally, when reviewing the studies included there was a large variety in consensus methods used and how well these methods were described [71].

## Conclusions

We found that studies on non-specific LBP, agreed in general on which symptoms (i.e., pain in lower back) and signs (i.e., absence of red flags) constitute a case definition, while considerable heterogeneity was found for the other MSDs. This scoping review can serve as a starting point for systematic reviews in disease specific case definitions with an initial broader inclusion process followed by a data synthesis of included studies with low risk of bias in order to distill the best evidence. But also, for future research in which expert consensus can be reached on a disorder/disease-specific case definition for a specific setting, such as patient care or occupational health research, and given a specific purpose, such as treatment or prevention of these (work-related) MSDs. For prevention purposes, case definitions on work-related MSDs should also include work-related exposure criteria.

## Supplementary Information

**Additional file 1.** Search strategy.

**Additional file 2.** Reasons for excluding studies based on full text.

**Additional file 3.** Study characteristics and MSD case definitions of included studies.

## Data Availability

All data generated or analysed during this study have been included in this published article.

## Abbreviations

MSDs: Musculoskeletal disorders
LBP: Low back pain
LRS: Lumbosacral radicular syndrome
SAPS: Subacromial pain syndrome
CTS: Carpal tunnel syndrome
OA: Osteoarthritis
OMEGA-NET: Network on the Coordination and Harmonisation of European Occupational Cohorts
EU COST: European Cooperation in Science and Technology
ICOH: International Commission of Occupational Health
PRISMA-ScR: Systematic Reviews - extension for Scoping Reviews
MRI: Magnetic Resonance Imaging
ICD-11: The International Classification of Diseases
WHO: World Health Organisation
