# Supplementary material for: Diagnostic criteria for musculoskeletal disorders for use in occupational healthcare or research: a scoping review of consensus- and synthesised-based case definitions
Source: BMC Musculoskelet Disord. 2021 Feb 11;22:169. doi: 10.1186/s12891-021-04031-z (PMC7879660; doi:10.1186/s12891-021-04031-z)
Supplement: Supplementary file 1 — Additional file 1. Search strategy. [file 12891_2021_4031_MOESM1_ESM.docx]

**Additional file 1 Search strategy**

| **Ovid MEDLINE(R) ALL <1946 to June 25, 2020> Search date: 26 June 2020** |  |
| --- | --- |
| **Searches** | **Results** |
| delphi technique/ or consensus/ or exp "consensus development conferences as topic"/ or "guidelines as topic"/ or "practice guidelines as topic"/ | 173348 |
| (consensus or guideline?).kf,ti. | 108529 |
| delphi.ab,kf,ti. | 10876 |
| (consensus development conference or consensus development conference nih or guideline or practice guideline).pt. | 43477 |
| ((expert? or committee? or society) adj5 (question* or inventor* or agreement?)).ab,kf,ti. | 6450 |
| ((definition? or framework? or model or criteria) adj3 (develop* or assess* or evaluat*)).ab,kf,ti. | 200656 |
| or/1-6 [consensus documents] | 459606 |
| low back pain/ | 21728 |
| ((low* back adj3 (pain? or ach*)) or lumbago).mp. [mp=title, abstract, original title, name of substance word, subject heading word, floating sub-heading word, keyword heading word, organism supplementary concept word, protocol supplementary concept word, rare disease supplementary concept word, unique identifier, synonyms] | 37979 |
| Lumbosacral radicular syndrome.ab,kf,ti. | 51 |
| rotator cuff injuries/ | 5728 |
| (rotator cuff adj3 tear*).mp. [mp=title, abstract, original title, name of substance word, subject heading word, floating sub-heading word, keyword heading word, organism supplementary concept word, protocol supplementary concept word, rare disease supplementary concept word, unique identifier, synonyms] | 5602 |
| shoulder pain/ and rotator cuff/ | 547 |
| shoulder impingement syndrome/ | 1764 |
| ((Subacromial or shoulder?) adj2 impingement).mp. [mp=title, abstract, original title, name of substance word, subject heading word, floating sub-heading word, keyword heading word, organism supplementary concept word, protocol supplementary concept word, rare disease supplementary concept word, unique identifier, synonyms] | 2403 |
| carpal tunnel syndrome/ | 8588 |
| (carpal tunnel syndrome or (carpal tunnel and neuropath*)).mp. [mp=title, abstract, original title, name of substance word, subject heading word, floating sub-heading word, keyword heading word, organism supplementary concept word, protocol supplementary concept word, rare disease supplementary concept word, unique identifier, synonyms] | 10991 |
| exp elbow tendinopathy/ | 1690 |
| (elbow tendinopath* or tennis elbow?).mp. [mp=title, abstract, original title, name of substance word, subject heading word, floating sub-heading word, keyword heading word, organism supplementary concept word, protocol supplementary concept word, rare disease supplementary concept word, unique identifier, synonyms] | 2101 |
| "osteoarthritis, knee"/ | 19823 |
| (knee? adj3 osteoarthr*).mp. [mp=title, abstract, original title, name of substance word, subject heading word, floating sub-heading word, keyword heading word, organism supplementary concept word, protocol supplementary concept word, rare disease supplementary concept word, unique identifier, synonyms] | 26881 |
| "osteoarthritis, hip"/ | 8566 |
| ((hip? adj3 osteoarthr*) or coxarthr*).mp. [mp=title, abstract, original title, name of substance word, subject heading word, floating sub-heading word, keyword heading word, organism supplementary concept word, protocol supplementary concept word, rare disease supplementary concept word, unique identifier, synonyms] | 12459 |
| or/8-23 [selected MSD's] | 97256 |
| 7 and 24 | 2672 |
| limit 25 to yr="2000-current" | 2401 |
| animals/ not humans/ | 4677648 |
| 26 not 27 | 2349 |
|  |  |
|  |  |
| **Web of Science Search date: 26 June 2020** |  |
| **Searches** | **Results** |
| TS=(delphi or consensus or guideline?) | 654268 |
| TS=((expert? or committee? or society) NEAR/4 (question* or inventor* or agreement?)) | 5416 |
| TS=((definition? or framework? or model or criteria) NEAR/2 (develop* or assess* or evaluat*)) | 576171 |
| #1 OR #2 OR #3 | 1218260 |
| TS=((("lower back" or "low back") NEAR/2 (pain? or ach*)) or lumbago) | 688 |
| TS=("Lumbosacral radicular syndrome") | 40 |
| TS=("rotator cuff" NEAR/2 tear*) | 5832 |
| TS=("shoulder pain" and "rotator cuff") | 1509 |
| TS=((Subacromial or shoulder?) NEAR/1 impingement) | 1125 |
| TS=(carpal tunnel syndrome or (carpal tunnel and neuropath*)) | 9799 |
| TS=("elbow tendinopathy" or "elbow tendinopathies" or "tennis elbow") | 1413 |
| TS=(knee NEAR/2 osteoarthritis) | 25655 |
| TS=((hip NEAR/2 osteoarthritis) or coxarthr*) | 6974 |
| #5 OR #6 OR #7 OR #8 OR #9 OR #10 OR #11 OR #12 OR #13 | 49452 |
| #4 AND #14 | 2629 |
| Limits: 2000-2020 \| WOS.ISSHP OR WOS.ISTP | 55 |
