# Supplementary material for: Diagnostic criteria for musculoskeletal disorders for use in occupational healthcare or research: a scoping review of consensus- and synthesised-based case definitions
Source: BMC Musculoskelet Disord. 2021 Feb 11;22:169. doi: 10.1186/s12891-021-04031-z (PMC7879660; doi:10.1186/s12891-021-04031-z)
Supplement: Supplementary file 2 — Additional file 2. Reasons for excluding studies based on full text. [file 12891_2021_4031_MOESM2_ESM.docx]

**Additional file 2 Reasons for excluding studies based on full text**

| **Article** | | **Reason for exclusion** | **No. papers** |
| --- | --- | --- | --- |
| 1 | Diercks RL. [Practice guideline ‘Diagnosis and treatment of the subacromial pain syndrome’]. Ned Tijdschr Geneeskd. 2014;158:A6985. | Duplicate in other language | 1 |
| 2 | Billis E, McCarthy CJ, Gliatis J, Stathopoulos I, Papandreou M, Oldham JA. Which are the most important discriminatory items for subclassifying non-specific low back pain. J Eval Clin Pract. 2010;16(3):542-9. | No case definition | 1 |
| 3 | Chou R, Qaseem A, Snow V, Casey D, Cross JT, Shekelle P, Owens DK. Diagnosis and treatment of low back pain: a joint clinical practice guideline from the American College of Physicians and the American Pain Society. Ann Intern Med. 2007;147(7):478-91. | No case definition | 2 |
| 4 | Dewitte V, De Pauw R, De Meulemeester K, Peersman W, Danneels L, Bouche K, et al. Clinical classification criteria for nonspecific low back pain: A Delphi-survey of clinical experts. Musculoskelet Sci Pract. 2018;34:66-76. | No case definition | 3 |
| 5 | Graham B, Regehr G, Wright JG. Delphi as a method to establish consensus for diagnostic criteria. J Clin Epidemiol. 2003;56(12):1150-6. | No case definition | 4 |
| 6 | Griffith LE, Hogg-Johnson S, Cole DC, Krause N, Hayden J, Burdorf A, et al. Low-back pain definitions in occupational studies were categorized for a meta-analysis using Delphi consensus methods. J Clin Epidemiol. 2007;60(6):625-33. | No case definition | 5 |
| 7 | Hunter DJ, Arden N, Conaghan PG, Eckstein F, Gold G, Grainger A, et al. Definition of osteoarthritis on MRI: results of a Delphi exercise. Osteoarthritis Cartilage. 2011;19(8):963-9. | No case definition | 6 |
| 8 | Nazarian LN, Jacobson JA, Benson CB, Bancroft LW, Bedi A, McShaneJM, et al. Imaging Algorithms for Evaluating Suspected Rotator Cuff Disease: Society of Radiologists in Ultrasound Consensus Conference Statement. Radiology. 2013;267(2):589-95. | No case definition | 7 |
| 9 | Nuckols T, Harber P, Sandin K, Benner D, Weng H, Shaw R, et al. Quality Measures for the Diagnosis and Non-Operative Management of Carpal Tunnel Syndrome in Occupational Settings. J Occup Rehabil. 2011;21(1):100-19. | No case definition | 8 |
| 10 | Oliva F, Piccirrilli E, Bossa M, Giai Via A, Colombo A, Chillemi C, et al. I.S.Mu.L.T - Rotator Cuff Tears Guidelines. Muscles Ligaments Tendons J. 2016;5(4):227-63. | No case definition | 9 |
| 11 | Puhl W, Bernau A, Böhle E, Brune K, Gerhardt P, Greitemann B, et al. [Ambulatory diagnosis and therapy of gonarthrosis]. Z Orthop Ihre Grenzgeb. 2000;138(1):85-92. | No case definition | 10 |
| 12 | Robb G, Arroll B, Reid D, Goodyear-Smith F. Summary of an evidence-based guideline on soft tissue shoulder injuries and related disorders--Part 1: Assessment. J Prim Health Care. 2009;1(1):36-41. | No case definition | 11 |
| 13 | Sakellariou G, Conaghan PG, Zhang W, Bijlsma JWJ, Boyesen P, D'Agostino MA, et al. EULAR recommendations for the use of imaging in the clinical management of peripheral joint osteoarthritis. Ann Rheum Dis. 2017;76(9):1484-1494. | No case definition | 12 |
| 14 | Sambandam SN, Priyanka P, Gul A, Ilango B. Critical analysis of outcome measures used in the assessment of carpal tunnel syndrome. Int Orthop. 2008;32(4):497-504. | No case definition | 13 |
| 15 | Burton CL, Chesterton LS, Chen Y, van der Windt DA. Clinical Course and Prognostic Factors in Conservatively Managed Carpal Tunnel Syndrome: A Systematic Review. Arch Phys Med Rehabil. 2016;97(5):836-852.e1. | No case definition | 14 |
| 16 | Conaghan PG, Felson D, Gold G, Lohmander S, Totterman S, Altman R. MRI and non-cartilaginous structures in knee osteoarthritis. Osteoarthritis Cartilage. 2006;14 Suppl A:A87-94. | No case definition | 15 |
| 17 | Graham B, Dvali L, Regehr G, Wright JG. Variations in diagnostic criteria for carpal tunnel syndrome among Ontario specialists. Am J Ind Med. 2006;49(1):8-13. | No case definition | 16 |
| 18 | Haswell K, Gilmour J, Moore B. Clinical decision rules for identification of low back pain patients with neurologic involvement in primary care. Spine. 2008;33(1):68-73. | No case definition | 17 |
| 19 | Hegmann KT, Hoffman HE, Belcourt RM, Byrne K, Glass L, Melhorn JM, et al. ACOEM practice guidelines: elbow disorders. J Occup Environ Med. 2013;55(11):1365-74. | No case definition | 18 |
| 20 | Lubowitz JH, Provencher MT, Poehling GG. ISAKOS Upper Extremity Committee Shoulder Rotator Cuff Consensus, Plus Chondrocyte Implantation and ACL Systematic Reviews. Arthroscopy. 2013;29(11):1733-4. | No case definition | 19 |
| 21 | Keith MW, Masear V, Chung KC, Maupin K, Andary M, Amadio PC, et al. American Academy of Orthopaedic Surgeons Clinical Practice Guideline on diagnosis of carpal tunnel syndrome. J Bone Joint Surg Am. 2009;91(10):2478-9. | No case definition | 20 |
| 22 | Lane LB, Starecki M, Olson A, Kohn N. Carpal tunnel syndrome diagnosis and treatment a survey of members of the American Society For Surgery of the Hand. J Hand Surg Am. 2014;39(11):2181-87.e4. | No case definition | 21 |
| 23 | Laštovková A, Nakládalová M, Fenclová Z, Urban P, Gad'ourek P, Lebeda T, et al. Low-Back Pain Disorders as Occupational Diseases in the Czech Republic and 22 European Countries. Cent Eur J Public Health. 2015;23(3):244-51. | No case definition | 22 |
| 24 | Lee J, Gupta S, Price C, Baranowski AP. Low back and radicular pain: a pathway for care developed by the British Pain Society. Br J Anaesth. 2013;111(1):112-20. | No case definition | 23 |
| 25 | Mooar PA, Doherty WJ, Murray JN, Pezold R, Sevarino KS. Management of Carpal Tunnel Syndrome. J Am Acad Orthop Surg. 2018;26(6):e128-e130. | No case definition | 24 |
| 26 | Murray J, Gross L. Optimizing the management of full-thickness rotator cuff tears. J Am Acad Orthop Surg. 2013;21(12):767-71. | No case definition | 25 |
| 27 | Nijs J, Apeldoorn A, Hallegraeff H, Clark J, Smeets R, Malfliet A, et al. Low back pain: guidelines for the clinical classification of predominant neuropathic, nociceptive, or central sensitization pain. Pain Physician. 2015;18(3):E333-46. | No case definition | 26 |
| 28 | Patel ND, Broderick DF, Burns J, Deshmukh TK, Fries IB, Harvey HB, et al. ACR Appropriateness Criteria Low Back Pain. J Am Coll Radiol. 2016;13(9):1069-78. | No case definition | 27 |
| 29 | Peter WF, Jansen MJ, Hurkmans EJ, Bloo H, Dekker J, Dilling RG, et al. Physiotherapy in hip and knee osteoarthritis development of a practice guideline concerning initial assessment, treatment and evaluation. Acta Reumatol Port. 2011;36(3):268-81. | No case definition | 28 |
| 30 | Pincus T, Santos R, Breen A, Burton AK, Underwood M. A review and proposal for a core set of factors for prospective cohorts in low back pain: a consensus statement. Arthritis Rheum. 2008;59(1):14-24. | No case definition | 29 |
| 31 | Rillo O, Riera H, Acosta C, Liendo V, Bolaños J, Monterola L, et al. PANLAR Consensus Recommendations for the Management in Osteoarthritis of Hand, Hip, and Knee. J Clin Rheumatol. 2016;22(7):345-54. | No case definition | 30 |
| 32 | Sandin KJ, Asch SM, Jablecki CK, Kilmer DD, Nuckols TK. Clinical quality measures for electrodiagnosis in suspected carpal tunnel syndrome. Muscle Nerve. 2010;41(4):444-52. | No case definition | 31 |
| 33 | Ariani A, Manara M, Fioravanti A, Iannone F, Salaffi F, Ughi N, et al. The Italian Society for Rheumatology clinical practice guidelines for the diagnosis and management of knee, hip and hand osteoarthritis. Reumatismo. 2019;71(S1):5-21. | No case definition | 32 |
| 34 | Doiron-Cadrin P, Lafrance S, Saulnier M, Cournoyer E, Roy JS, Dyer JO, et al. Shoulder Rotator Cuff Disorders: A Systematic Review of Clinical Practice Guidelines and Semantic Analyses of Recommendations. Arch Phys Med Rehabil. 2020;101(7):1233-1242. | No case definition | 33 |
| 35 | French SD, Nielsen M, Hall L, Nicolson PJA, van Tulder M, Bennell KL, et al. Essential key messages about diagnosis, imaging, and self-care for people with low back pain: a modified Delphi study of consumer and expert opinions. Pain. 2019;160(12):2787-2797. | No case definition | 34 |
| 36 | Kreiner DS, Matz P, Bono CM, Cho CH, Easa JE, Ghiselli G, et al. Guideline summary review: an evidence-based clinical guideline for the diagnosis and treatment of low back pain. Spine J. 2020;20(7):998-1024. | No case definition | 35 |
| 37 | Rahyussalim AJ, Zufar MLL, Kurniawati T. Significance of the Association between Disc Degeneration Changes on Imaging and Low Back Pain: A Review Article. Asian Spine J. 2020;14(2):245-257. | No case definition | 36 |
| 38 | Roux CH. Hip osteoarthritis guidelines differences, appicability and application? Joint Bone Spine. 2020;87(2):111-114. | No case definition | 37 |
| 39 | van Wambeke P, Desomer A, Jonckheer P, Depreitere B. The Belgian national guideline on low back pain and radicular pain: key roles for rehabilitation, assessment of rehabilitation potential and the PRM specialist. Eur J Phys Rehabil Med. 2020;56(2):220-227. | No case definition | 38 |
| 40 | Assmus H, Schwerdtfeger K, Wüstner-Hofmann M, Selbmann HK. [Development and implementation of the guideline "Diagnosis of and Therapy for Carpal Tunnel Syndrome"]. Handchir Mikrochir Plast Chir. 2007;39:289–92. | No consensus approach | 1 |
| 41 | Bernstein IA, Malik Q, Carville S. Low back pain and sciatica: summary of NICE guidance. BMJ 2017;356:i6748. | No consensus approach | 2 |
| 42 | Cook C, Hegedus E. Diagnostic utility of clinical tests for spinal dysfunction. Man Ther. 2011;16(1):21-5. | No consensus approach | 3 |
| 43 | Lötters F, Burdorf A, Kuiper J, Miedema H. Model for the work-relatedness of low-back pain. Scand J Work Environ Health. 2003;29(6):431-40. | No consensus approach | 4 |
| 44 | Chen WH, Liu XX, Tong PJ, Zhan HS. Diagnosis and management of knee osteoarthritis: Chinese medicine expert consensus (2015). Chin J Integr Med. 2016;22(2):150-3. | No consensus approach | 5 |
| 45 | Chen YT, Williams L, Zak MJ, Fredericson M. Review of Ultrasonography in the Diagnosis of Carpal Tunnel Syndrome and a Proposed Scanning Protocol. J Ultrasound Med. 2016;35(11):2311-2324. | No consensus approach | 6 |
| 46 | Goldfarb CA. The Clinical Practice Guideline on Carpal Tunnel Syndrome and Workers' Compensation. J Hand Surg Am. 2016;41(6):723-5. | No consensus approach | 7 |
| 47 | Katz JN, Simmons BP. Clinical practice. Carpal tunnel syndrome. N Engl J Med. 2002;346(23):1807-12. | No consensus approach | 8 |
| 48 | Lane NE. Clinical practice. Osteoarthritis of the hip. N Engl J Med. 2007;357(14):1413-21. | No consensus approach | 9 |
| 49 | Metzger RL. Evidence-based practice guidelines for the diagnosis and treatment of lumbar spinal conditions. Nurse Pract. 2016;41(12):30-37. | No consensus approach | 10 |
| 50 | Misso ML, Pitt VJ, Jones KM, Barnes HN, Piterman L, Green SE. Quality and consistency of clinical practice guidelines for diagnosis and management of osteoarthritis of the hip and knee: a descriptive overview of published guidelines. Med J Aust. 2008;189(7):394-9. | No consensus approach | 11 |
| 51 | Pal B. Diagnosis of carpal tunnel syndrome. Rheumatology. 2001;40(5):595-7. | No consensus approach | 12 |
| 52 | Runhaar J, Kloppenburg M, Boers M, Bijlsma H, Bierma-Zeinstra S. Towards developing diagnostic criteria for early hip osteoarthritis. Osteoarthritis and Cartilage. 2020;28:S39. | No full text | 1 |
| 53 | Runhaar J, Kloppenburg M, Boers M, Bijlsma H, Bierma-Zeinstra S. Towards developing diagnostic criteria for early knee osteoarthritis. Osteoarthritis and Cartilage. 2020;28:S385-S386. | No full text | 2 |
| 54 | Grazio S, Curković B, Vlak T, Kes VB, Jelić M, Buljan D, et al. [Diagnosis and conservative treatment of low back pain: review and guidelines of the Croatian Vertebrologic Society]. Acta Med Croatica. 2012;66(4):259-94. | Not eligible language | 1 |
| 55 | Guevara-López U, Covarrubias-Gómez A, Elías-Dib J, Reyes-Sánchez A, Rodríguez-Reyna TS, et al. Practice guidelines for the management of low back pain. Consensus Group of Practice Parameters to Manage Low Back Pain. Cir Cir. 2011;79(3):264-79, 286-302. | Not eligible language | 2 |
| 56 | Krawczyk-Szulc P, Wągrowska-Koski E, Puzder A, Markowski P, Walusiak-Skorupa J. [Diagnostic guidlines for occupational epicondylitis]. Med Pr. 2015;66(3):443-50. | Not eligible language | 3 |
| 57 | Lepola V, Paloneva J, Huuskonen M, Kallinen M, Karppinen J, Mattila K, et al. [Update on current care guidelines. The tendon disorders of the shoulder]. Duodecim. 2015;131(2):194-5. | Not eligible language | 4 |
| 58 | Todnem K, Sand T. [Neurography for diagnosing carpal tunnel syndrome]. Tidsskr Nor Laegeforen. 2013;133(2):170-3. | Not eligible language | 5 |
| 59 | Crossley KM, Stefanik JJ, Selfe J, Collins NJ, Davis IS, Powers CM, et al. 2016 Patellofemoral pain consensus statement from the 4^th^ International Patellofemoral Pain Research Retreat, Manchester. Part 1: Terminology, definitions, clinical examination, natural history, patellofemoral osteoarthritis and patient-reported outcome measures. Br J Sports Med. 2016;50(14):839-43. | Not eligible outcome | 1 |
| 60 | Fairbank J, Gwilym SE, France JC, Daffner SD, Dettori J, Hermsmeyer J, Andersson G. The role of classification of chronic low back pain. Spine. 2011;36(21 Suppl):S19-42. | Not eligible outcome | 2 |
| 61 | Amini B, Beckmann NM, Beaman FD, Wessell DE, Bernard SA, Cassidy RC, et al. ACR Appropriateness Criteria ® Shoulder Pain-Traumatic. J Am Coll Radiol. 2018;15(5S):S171-S188. | Not eligible outcome | 3 |
| 62 | Rubin DA, Roberts CC, Bencardino JT, Bell AM, Cassidy RC, Chang EY, et al. ACR Appropriateness Criteria ® Chronic Wrist Pain. J Am Coll Radiol. 2018;15(5S):S39-S55. | Not eligible outcome | 4 |
| 63 | Favard L, Berhouet J, Walch G, Chaoui J, Lévigne C. Superior glenoid inclination and glenoid bone loss: Definition, assessment, biomechanical consequences, and surgical options. Orthopade. 2017;46(12):1015-1021. | Not eligible outcome | 5 |
| 64 | Resnick DK, Malone DG, Ryken TC. Guidelines for the use of discography for the diagnosis of painful degenerative lumbar disc disease. Neurosurg Focus. 2002;13(2):E12. | Not eligible outcome | 6 |
| 65 | Tomkins-Lane C, Melloh M, Lurie J, Smuck M, Battié MC, Freeman B, et al. ISSLS Prize Winner: Consensus on the Clinical Diagnosis of Lumbar Spinal Stenosis: Results of an International Delphi Study. Spine. 2016;41(15):1239-46. | Not eligible outcome | 7 |
