# Supplementary material for: Diagnostic criteria for musculoskeletal disorders for use in occupational healthcare or research: a scoping review of consensus- and synthesised-based case definitions
Source: BMC Musculoskelet Disord. 2021 Feb 11;22:169. doi: 10.1186/s12891-021-04031-z (PMC7879660; doi:10.1186/s12891-021-04031-z)
Supplement: Supplementary file 3 — Additional file 3. Study characteristics and MSD case definitions of included studies. [file 12891_2021_4031_MOESM3_ESM.docx]

| **Additional file 3 Study characteristics and MSD case definitions of included studies** | | | | | | | | |
| --- | --- | --- | --- | --- | --- | --- | --- | --- |
| **Study characteristics** | | | | | | **MSD case definition** | | |
| **MSD category** | **Study and publication year** | **Country** | **Expert consensus method or synthesis method** | **Involved disciplines** | **Study aim** | **Symptoms** | **Signs** | **Instrumental (imaging)** |
| Non-specific LBP | Koes et al. 2001 [38] | Nether-lands | Systematic review | NA | Clinical | History taking (e.g. pain). | Physical examination (e.g. widespread neurologic changes). | Radiographs not useful for non-specific LBP. |
|  | Koes et al. 2004 [36] | Nether-lands | NR^1^ | Epidemiologist, orthopaedic, general practitioner, physical therapist (e.g. chiropractor), medical technology assessment, rheumatology, insurance medicine, rehabilitation medicine, psychologist, occupational physician, Neurosurgery, radiologist, anaesthesiology, patients’ associations. | Clinical | < 12 weeks of pain, muscle tension or stiffness in lower back without leg pain. | Absence of physical cause (e.g. malignity). | NR |
|  | Koes et al. 2006 [39] | Nether-lands | NR | General practitioner and health technology assessment. | Clinical | < 3 months of pain, muscle tension or stiffness localized below the costal margin and above the inferior gluteal folds, without leg pain. The most important symptoms of non-specific low back pain are pain and disability. | Physical examination. | Imaging is not recommended. |
|  | Negrini et al. 2006 [46] | Italy | NR | Neurosurgery, general medicine, orthopaedics, rheumatology, physical and rehabilitation medicine, work medicine, industrial hygiene, physiotherapist. | Clinical | A pain with/without functional limitation, lasting less than 4 weeks (1 month), in the posterior region included between the inferior limit of the costal arch and the inferior buttock fold, possibly with posterior irradiation to the thigh, but not below the knee. | NR | NA |
|  | Dionne et al. 2008 [29] | Inter-national | Modified Delphi^3,4^ | Back pain experts: epidemiology and biostatistics, chiropractor, public health, preventive medicine, sport science, biomechanics, occupational health, general medicine, rehabilitation, anaesthesiology, pain, clinical pharmacology and toxicology, extramural medicine, psycho-social and disability research, veterans affairs healthcare system. | Research | Pain in low back. Pain bad enough to limit usual activities or change your daily routine for more than one day. | NR | NA |
|  | Dagenais et al. 2010 [26] | USA | Systematic review | NA | Clinical | Back pain. | No sign of a serious spinal pathology, specific causes of LBP and substantial neurologic involvement. No red flags. | Only when potentially serious spinal pathology or specific causes are present. |
|  | Koes et al. 2010 [37] | Nether-lands | Systematic review | NA | Clinical | Low back pain < 12 weeks. | The exclusion of specific diseases by means of physical examination. | Routine imaging not indicated. |
|  | Laerum et al. 2010 [40] | Norway | Systematic review | Physicians: orthopaedics, neurology, surgery, general medicine. | Clinical | History taking (e.g. pain < 12 weeks). | Neurological examination: Lasegue’s test and crossing Lasegue’s test. | MRI of the lumbosacral column after 4–6 weeks when no signs of improvement. |
|  | O’Connell et al. 2016 [47] | USA, UK and Canada | Narrative review | Physiotherapist, pain medicine, clinical guideline development for management of LBP. | Clinical | Pain in the low back. | No identifiable cause and no clear association with a specific, serious underlying anatomical impairment or disease process. | NR |
|  | Chenot et al. 2017 [24] | Ger-many | Clinical practice guideline (systematic review and formal consensus process) | Insurance medicine, psychotherapy, general practitioner, anaesthesiology, occupational and environmental medicine, surgeon, experimental and clinical pharmacology and toxicology, internal medicine, manual medicine, neurosurgery, neurology, neuro rehabilitation, orthopaedics, surgeon, physical medicine and rehabilitation, psychology, rheumatology, trauma surgery, radiology, pain society, occupational therapy, phytotherapy. | Clinical | Acute or recurrent low back pain. | No sign of a dangerous course or other serious conditions. | Without "red flags" patients should not undergo any imaging. |
|  | Oliveira et al. 2018 [48] | Inter-national | Review of clinical guidelines | NR | Clinical | Low back pain. | Not attributable to a known cause. | Imaging not recommended. |
|  | Reith, 2020 [51] | Ger-many | Guideline | 29 professional societies and organisations. | Clinical | Detailed medical history (e.g. pain). | Absence of specific spinal diseases by means of physical examination. | Imaging not recommended. |
| LRS/  sciatica | Koes et al. 2001 [38] | Nether-lands | Systematic review | NA | Clinical | History taking (e.g. pain). | Physical examination including straight leg raising test. | Radiographs. |
|  | Koes et al. 2006 [39] | Nether-lands | NR | General practitioner and health technology assessment. | Clinical | Pain, muscle tension, or stiffness localised below the costal margin and above the inferior gluteal folds with leg pain. | Straight leg raising test induces more leg pain. | Imaging is not recommended. |
|  | Negrini et al. 2006 [46] | Italy | NR | Neurosurgery, general medicine, orthopaedics, rheumatology, physical and rehabilitation medicine, work medicine and industrial hygiene, physiotherapist. | Clinical | A pain with/without functional limitation, lasting less than 4 weeks (1 month), in the posterior region included between the inferior limit of the costal arch and the inferior buttock fold, with posterior irradiation below the knee or anterior to the thigh. Leg pain can be present even without lumbar pain. | NR | NA |
|  | Cid et al. 2015 [25] | Spain | Modified Delphi^3,4^ | Experts in pain treatment (e.g. anaesthesiology rehabilitation and internal medicine). | Clinical | Radicular pain in 1 lower limb. | One or more positive neurological test(s) indicating nerve root irritation or neurological deficit (e.g. a positive Lasègue’s test at 60°). | To attribute the pain aetiology to an MRI-diagnosed disk hernia, there must  be a clinical correlation with the symptoms. |
|  | Verburg et al. 2015 [55] | Nether-lands | Expert group^3^ | Neurologist, neurosurgeon, orthopaedics, physical therapist, general practitioner, epidemiologist. | Clinical | Radiating pain in 1 lower limb, irritation symptoms, and | Finger-floor distance of > 25 cm, Lasègue’s test, and crossing Lasegue’s test, and neurological signs | Imaging not recommended. |
|  | Genevay et al. 2017 [31] | Switzer-land | Delphi^2,3,4^ and cohort study | Spine specialists: rheumatologist, internal medicine, physiotherapist, clinical epidemiology /chiropractic back pain, physical & rehabilitation medicine, sports medicine, occupational medicine. | Research | Monoradicular leg pain. | 1. Straight leg raise test ≤ 60° or positive femoral stretch test.  2. Unilateral ankle reflex decrease.  3. Unilateral muscle weakness  4. Unilateral patient-reported pain in legs | NR |
| Chronic LBP | Koes et al. 2001 [38] | Nether-lands | Systematic review | NA | Clinical | History taking (e.g. pain in low back) | Physical examination including straight leg raising test | NR |
|  | Koes et al. 2004 [36] | Nether-lands | NR | Epidemiologist, orthopaedic, general practitioner, physical therapist (e.g. chiropractor), medical technology assessment, rheumatology, insurance medicine, rehabilitation medicine, psychologist, occupational physician, neurosurgery, radiologist, anaesthesiology, patients’ associations. | Clinical | > 12 weeks pain, muscle tension or stiffness in lower back with or without leg pain. | Absence of physical cause (e.g. malignity). | NR |
|  | Koes et al. 2006 [39] | Nether-lands | NR | General practitioner and health technology assessment. | Clinical | > 6 weeks of pain, muscle tension, or stiffness localized below the costal margin and above the inferior gluteal folds, without leg pain. The most important symptoms of non-specific low back pain are pain and disability. | Physical examination including straight leg raising test. | Imaging is not recommended |
|  | Negrini et al. 2006 [46] | Italy | NR | Neurosurgery, general medicine, orthopaedics, rheumatology, physical and rehabilitation medicine, work medicine and industrial hygiene, physiotherapist. | Clinical | Pain, with/without functional limitation, lasting more than 3 months, in the posterior region included between the inferior limit of the costal arch and the inferior buttock fold, possibly with posterior irradiation to the thigh, but not below the knee. | NR | NA |
|  | Koes et al. 2010 [37] | Nether-lands | NR | NR | Clinical | Low back pain > 12 weeks. | The exclusion of specific diseases by means of physical examination including straight leg raising test. | Routine imaging not indicated |
|  | Laerum et al. 2010 [40] | Norway | Systematic review | orthopaedics, neurology and surgery, general medicine. | Clinical | Duration over 3 months. | Neurological examination: Lasègue’s test and crossing Lasègue’s test. | MRI of the lumbosacral column after 4–6 weeks when no signs of improvement. |
|  | Stanton et al. 2011 [53] | Austra-lia | Modified Delphi^3.4^ | International experts in the area of LBP (not further specified). | Research | A return of LBP lasting at least 24hrs with a pain intensity of >2 on an 11-point NRS (>20mm on a 100mm VAS) following a period of at least 30 days pain-free. | NR | NA |
|  | Deyo et al. 2014 [28] | USA | Expert group^3,4^ | Primary care, chronic pain, psychology, orthopaedic spine surgery, rheumatology, musculoskeletal radiology, internal medicine, physical therapy, chiropractic care, epidemiology, neurosurgery, functional brain imaging, spine rehabilitation, geriatric medicine. | Research | >3 months LBP problem for individual for at least half the days in the past 6 months. | NR | NA |
|  | Oliveira et al. 2018 [48] | Inter-national | Review of clinical guidelines | NR | Clinical | Low back pain > 12 weeks. | Not attributable to a known physical cause. | Imaging not recommended. |
|  | Ma et al. 2019 [43] | China | NR | NR | Clinical | Pain or discomfort originating from the waist, which lasts for at least 12 weeks, but no radiculopathy or specific spinal diseases. | Absence of specific spinal diseases by means of physical examination. | Imaging not recommended. |
| SAPS | Sluiter et al. 2001 [7] | Nether-lands | Review/  Expert group^3^ | Experts on work-related upper-extremity musculoskeletal disorders | Clinical | 1. Intermittent shoulder pain without  paraesthesia.  2. Pain worsened by active elevation movement of the upper arm as in scratching of the upper back.  Symptoms present now or on at least 4 days during the last 7 days. | At least one of the following tests positive: 1. Resisted shoulder abduction, external rotation, or internal rotation. 2. Resisted elbow flexion. 3. Painful arc on active upper arm elevation. | NA |
|  | Alqunaee et al. 2012 [20] | Ireland | Systematic review with meta-analysis | Surgeon, physical therapist, general practitioner. | Clinical | Shoulder pain and/or weakness (depending on test). | 1. Pain tested with:  a. Neer's sign test.  b. Hawkins-Kennedy test.  c. Drop arm sign.  2. Weakness tested with:  a. Empty can test.  b. Drop arm sign.  c. Lift-off test (highest diagnostic utility). | NR |
|  | Diercks et al. 2014 [13] | Nether-lands | Review | Orthopaedic , physical therapy, general practitioner, rehabilitation medicine, occupational medicine, radiology. | Clinical | Shoulder pain, localised around the acromion, often worsening during or subsequent to lifting of the arm. | SAPS:  a combination of the:  1. Hawkins-Kennedy test.  2. The painful arc test.  3. Infraspinatus muscle strength test.  Rotator cuff tear:  1. The drop-arm test and the infraspinatus and supraspinatus muscle strength | Diagnostic imaging is  useful after 6 weeks of symptoms. Ultrasound examination is  the recommended imaging, to exclude a rotator cuff rupture. |
|  | Eubank et al. 2016 [30] | Canada | Modified Delph^3,4^ | Athletic therapy, physiotherapy, sport medicine, and orthopaedic surgery. | Clinical | 1. Shoulder pain (incl. night pain, pain at rest).  2. Stiffness.  3. Loose or unstable shoulder.  4. Weakness.  5. Painful clicking, grinding, or clunking.  6. Unusual sensations such as catching, locking, or grinding. | 1. Dislocation.  2. Shoulder comes out of place.  3. Range of motion assessed with painful arc test. | X-ray / Ultrasound according to algorithm. |
|  | Schumaier et al. 2020 [52] | USA | Delphi study^2,3,4^ | Surgeons | Clinical | NR | Retraction of tendon(s) to the glenoid rim, measured in either the coronal or axial plane, and/or  ≥67% of the greater tuberosity exposed, measured in the  sagittal plane, diagnosed either with MRI or intraoperatively. | MRI |
| CTS | Sluiter et al. 2001 [7] | Nether-lands | Review/  Expert group^3^ | Experts on work-related upper-extremity musculoskeletal disorders. | Clinical | Intermittent paraesthesia or (night)pain in at least 2 of digits I, II or III; either may be present at night as well (allowing pain in the palm, wrist, or radiation proximal to the wrist).  Symptoms present now or on at least 4 days during the last 7 days. | Positive on at least one of the following tests: 1. Flexion compression test.  2. Carpal compression test.  3. Tinel’s sign. 4. Phalen’s test. 5. Two-point discrimination test. 6. Resisted thumb abduction or motor loss with wasting of abductor pollicis brevis muscle. | NA |
|  | Graham et al. 2006 [33] | Canada | Delphi technique/  Validation^3^ | Experts (not further specified) | Clinical | 1. Numbness and tingling in the median nerve distribution.  2. Nocturnal numbness.  3. Weakness/atrophy of the thenar musculature. | Loss of 2-point discrimination on:  1. Tinel’s sign.  2. Phalen’s test. | NA |
|  | Assmus et al. 2007 [22] | Ger-many | Delphi technique^3^ | Hand surgery, neurosurgery, neurology, orthopaedics, clinical neurophysioIogy, functional imaging, plastic, reconstructive and aesthetic surgery, and surgery for traumatology. | Clinical | 1. Brachialgia paraesthetica nocturne (sleeping feeling in the hand).  2. "Electric shocks" by gripping movements or a permanent persistent sensation "the fingers tingle constantly". | 1. Motor neurography:  a. Distal motor latency: at a distance of 6,5cm is a value of > 4,2ms pathologic.  2. Sensitive neurography:  a. Difference in nerve conduction speed > 8 m/s between N. medianus and N.ulnaris.  b. Comparison of the sensitive potential on the ring finger – latency difference of 0,5ms between N.medianus and N.ulnaris. | Electrodiagnostic findings (nerve conduction velocity studies). |
|  | Keith et al. 2009 [35] | USA | Systematic review | Physicians (not further specified). | Clinical | Numbness along the distribution of the median nerve increasing at night when sleeping  improvement by shaking the hands exacerbation when driving or holding a telephone. | 1. Personal characteristics (e.g., age, sex, weight, height).  2. Range of motion of hand/wrist.  3. Observation of deformity, swelling, atrophy, skin trophic changes.  4. Pinch/grip strength.  5. Hand diagram.  6. Sensory examination (e.g., two-point discrimination, Semmes-Weinstein monofilament, vibrometry, texture discrimination).  7. Manual muscle testing of the upper extremity (e.g., examine for muscular atrophy, especially in the thenar muscle group)  8. Provocative tests (e.g., Phalen test, Tinel sign, median nerve compression test, reverse Phalen test)  9. Discriminatory examination for alternative diagnoses (e.g., radiculopathy, neuropathy, pain syndromes, arthritis, tendinitis, vascular abnormalities). | 1. Electrodiagnostic findings (nerve conduction velocity studies) recommended:  a. To differentiate among diagnoses.  b. In the presence of thenar atrophy and/or persistent numbness.  c. If clinical and/or provocative tests are positive and surgical management is being considered.  2. Not routinely evaluate patients suspected of having CTS with new technology such as magnetic resonance imaging (MRI), CT, and pressure-specified sensorimotor devices (PSSDs). |
|  | Geraets et al.2010 [32] | Nether-lands | NR | NR | Clinical | 1. (nocturnal, painful) tingling, and/or numbness of the thumb, index finger, middle finger, radial side of the ring finger and adjacent region of the palm.  2. Fluttering with hands can provide relief (‘flick-sign’)). | 1. Provocation tests (van Phalen of Tinel) are not recommended. | NA |
|  | Descatha et al. 2011 [27] | Inter-national | Literature review | NA | Research | Symptoms (e.g. numbness, tingling, pain). | Physical examination (e.g. Semmes-Weinstein sensory testing,  Tinel’s test, and Phalen’s manoeuvre) | Nerve conduction study. |
|  | Patijn et al. 2011 [49] | Nether-lands/  USA | Systematic review | Anaesthesiology | Clinical | 1. Paraesthesia, pain, and numbness in the hand.  2. Pain in hand, wrist and forearm.  3. Atypical localization of tingling sensations. | 1. Nerve conduction examinations of the nervus medianus at both hands. | MRI of the wrist does not provide additional diagnostic information in case of CTS. |
|  | Cartwright et al. 2012 [23] | USA | Systematic review | Physicians specializing in neurology, physical medicine and rehabilitation, and radiology. | Clinical | NR | 1. Cut-off > 8,5 mm2 of the median nerve cross-sectional area determined with ultra-sound | Neuromuscular ultrasound. |
| Elbow tendino-pathy | Sluiter et al. 2001 [7] | Nether-lands | Review/  Expert group^3^ | Experts on work-related upper-extremity musculoskeletal disorders. | Clinical | Intermittent, activity dependent pain directly located around the lateral or medial epicondyle.  Symptoms present now or on at least 4 days during the last 7 days | Local pain on resisted wrist extension (lateral) or on resisted wrist flexion (medial). | NA |
| Hip OA | Reijman et al. 2004 [50] | Nether-lands | Review | NA | Research | Hip pain.  Limited range of motion.  Disability.  Morning stiffness < 1 hour. | Minimal joint space of ≤ 1,5mm. | Radiograph. |
|  | Swierstra et al. 2009 [54] | Nether-lands | Guideline | physiotherapists, orthopaedics, radiologists, general practitioner, epidemiology, rheumatology, radiologist, nutrition epidemiologist. | Clinical | Hip pain | At least two of the three signs: 1. erythrocyte sedimentation rate ≤ 20 mm after 1 hour. 2. Femoral or acetabular osteophytes. 3. Joint space narrowing. | X-ray only recommended in the case of discrepancies between anamnesis and physical examination. |
|  | Leyland et al. 2018 [41] | International | Expert group^3,4^ | The panel consisted of multidisciplinary, geographically diverse experts on OA and population-based cohort studies. | Research | Hip pain. | Degeneration. | X-ray Kellgren and Lawrence grade ≥ 2. |
|  | Martel-Pelletier et al. 2018 [44] | France | Expert group^3,4^ | Rheumatologists, a physical and rehabilitation medicine specialist, a clinical scientist, general practitioner. | Clinical | (no abnormal) hip pain. | Radiographic joint space narrowing and/or osteophyte(s). No morphological anomalies | X-ray |
| Knee OA | Swierstra et al. 2009 [54] | Netherlands | Expert group | Physiotherapists, orthopaedics, radiologists, general practitioner, epidemiology, rheumatology, radiologist, nutrition epidemiologist. | Clinical | Pain in the knee and at least three of the six symptoms/signs/personal factors: 1. Age ≥ 50 years.  2. Stiffness < 30 minutes.  3. Crepitus.  4. Pain at palpation knee bone.  5. Bone deformation.  6. No palpable warmth. | | X-ray only recommended in the case of discrepancies between anamneses and physical examination. |
|  | Zhang et al. 2010 [56] | United King-dom | Systematic review/ delphi^3^ | OA experts (not further specified). | Clinical | Persistent knee pain.  Limited morning stiffness.  Reduced function.  Crepitus. | Restricted movement.  Bony enlargement. | As an adjunct:  plain radiographs. |
|  | Alshami et al. 2014 [21] | Saudi-Arabia | Narrative review | NA | Clinical | Knee joint pain.  Stiffness < 30 minutes.  Crepitus | Clinical:  1. Bony enlargement.  2. Bony tenderness.  3. No palpable warmth.  Laboratory:  1. Erythrocyte sedimentation rate <40 mm/hr.  2.Rheumatoid factor < 1:40.  3. Synovial fluid analysis; clear, viscous, white blood cell count < 2.000 µl (2.00 x 109 per L).  Radiographic:  1. Osteophytes and possible barrowing of joint space. | X-ray. |
|  | Migliore et al. 2017 [45] | Italy/  inter-national | Systematic review / delphi^4^ | Clinicians: rheumatologists.  Patients: all woman. Researchers: in basic science of OA) and specialists in rheumatology, internal medicine, physical medicine and epidemiology. | Clinical | (a) two mandatory symptoms (knee pain in the absence of any recent trauma or injury and very short joint stiffness, lasting for less than 10 min, when starting movement) even in the absence of risk factors, or  (b) knee pain, and 1 or 2 risk factors or  (c) three or more risk factors in the presence of at least one mandatory symptom, with symptoms lasting less than 6 months.  These criteria are applicable in the absence of active inflammatory arthritis, generalized pain, Kellgren- Lawrence grade >0, any recent knee trauma or injury, and age lower than 40 years.  Risk factors:  Overweight (body mass index >25).  Family history of OA.  Malalignment.  Lower limbs dissymmetry.  OA in other sites.  Metabolic syndrome.  Not being ready to run or walk fast after a period of inactivity. | | X-ray Kellgren- Lawrence grade >0 |
|  | Huang et al. 2018 [34] | China | Expert group | NR | Clinical | Knee pain.  Morning stiffness < 30 minutes.  Crepitus. | Recurrent episodes of knee arthralgia for 1 month.  Asymmetric arthrostenosis.  Subchondral bone sclerosis.  Cystic degeneration.  Osteophytes.  Synovial fluid; clear and viscous – leukocyte count < 2000 ml. | X-ray. |
|  | Leyland et al. 2018 [41] | Inter-national | Expert group^3,4^ | Multidisciplinary, geographically diverse experts on OA and population-based cohort studies. | Research | Knee pain. | Degeneration. | X-ray Kellgren and Lawrence grade ≥ 2. |
|  | Luyten et al. 2018 [42] | International | Expert group | Rheumatology, orthopaedic surgery, radiology, physiotherapy, epidemiology, health sciences, experimental research, basic and translational science related to OA. | Clinical | Two out of the 4 KOOS subscales need to score positive (≤85%):  1. Knee pain.  2. Stiffness and other symptoms.  3. Functioning, daily living.  4. Knee-related QOL.  At least one present out of joint line tenderness or crepitus.  Degeneration. | | X-ray Kellgren and Lawrence grade ≥ 2. |
|  | Martel-Pelletier et al. 2018 [44] | France | Expert group^3,4^ | The expert panel consisted of rheumatologists, a physical and rehabilitation medicine  specialist, a rheumatologist and physical and rehabilitation  medicine specialist, and a clinical scientist. | Clinical | Knee pain. | Joint space narrowing and/or osteophyte(s) and no effusion.  Absence of associated metabolic/inflammatory syndrome. | X-ray  No indication for an MRI**.** |

1. The authors reported the use of a consensus- or synthesised based method but did not report what type of consensus- or synthesised-based method.
2. Provided a definition of consensus on the case definition a priori.
3. Applied iteration.
4. Described how and which participants were selected.

Abbreviations: MSD (Musculoskeletal Disorder); NR (Not reported); NA (not applicable); Magnetic Resonance Imaging (MRI); NRS (Numeric Rating Scale); VAS (Visual Analogue Scale); LBP (Low Back Pain); SAPS (subacromial pain syndrome); CTS (carpal tunnel syndrome); PSSDs (Pressure-Specified Sensorimotor Devices); OA (osteoarthritis).
